# Clinical Outcome of Bortezomib Retreatment in Patients with Relapsed or Refractory Multiple Myeloma

**DOI:** 10.1155/2014/145843

**Published:** 2014-10-30

**Authors:** Jae-Sook Ahn, Sung-Hoon Jung, Seung-Shin Lee, Seo-Yeon Ahn, Deok-Hwan Yang, Yeo-Kyeoung Kim, Hyeoung-Joon Kim, Je-Jung Lee

**Affiliations:** Department of Hematology-Oncology, Chonnam National University Hwasun Hospital, 322 Seoyangro, Hwasun, Jeollanamdo 519-763, Republic of Korea

## Abstract

This retrospective study investigated the clinical efficacy and safety of bortezomib retreatment in patients with relapsed or refractory multiple myeloma (MM). A total of 30 patients who relapsed or progressed after ≥6 months since the last dose of their previous bortezomib therapy were included in this study. During the median 6 cycles (range: 2–12) of bortezomib retreatment, 10 (33.3%), 2 (6.7%), and 6 (20.0%) patients achieved complete response, very good partial response, and partial response, respectively. Grade 3 or 4 neutropenia (47.0%), thrombocytopenia (43.0%), anemia (10.0%), and peripheral sensory neuropathy (3.0%) were observed. The median time to progression, progression-free survival, and overall survival were 5.8 months (95% CI: 2.6–9.0), 5.5 months (95% CI: 4.2–6.8), and 13.4 months (95% CI: 6.1–20.7), respectively. Patients who received bortezomib retreatment ≥12 months from initial last therapy had a 1-year OS rate of 65.8% (95% CI: 43.5–88.1) while patients receiving retreatment after 6–12 months interval had a 1-year OS rate of 41.7% (95% CI: 13.9–69.5) (*P* = 0.038). In conclusion, this study demonstrates that retreatment with bortezomib is an effective strategy for patients with MM who relapsed at a long interval after initial bortezomib therapy.

## 1. Introduction

Over the past decade the introduction of novel agents, such as thalidomide, lenalidomide, and bortezomib, has advanced the treatment outcomes of patients with multiple myeloma (MM) [1, 2]. These agents are considered effective in controlling both newly diagnosed and relapsed MM. However, most patients with MM are observed to progress over various durations. The basic treatment strategy for relapsed or refractory MM is sequential drug treatment with untreated agents, because the disease is likely to have become resistant to the particular class of drug used for initial treatment [3].

Generally, relapse is thought to be due to the changing tumor biology and evolution of drug resistant phenotypes within the tumor [4, 5]. However, incorporation of novel agents into the first-line therapy raises new theories about the feasibility of retreatment with such agents. The factors influencing treatment choice in the relapsed setting include the duration of response to prior therapy and the associated toxicity profiles [6]. The National Comprehensive Cancer Network clinical guidelines recommend repeating the same primary induction therapy if the relapse occurs with an interval of ≥6 months after primary therapy [7]. Several studies reported the results of bortezomib retreatment in MM. In a prospective phase II study, retreatment with bortezomib resulted in an overall response rate (ORR) of 40%, time to progression (TTP) was 8.4 months, and grade 3-4 thrombocytopenia (35%) was the most common adverse event [6]. In retrospective reviews of relatively large numbers of patients, the ORR was 21–63.3% and TTP was 9.3 months [8–10]. One of the most important considerations for the choice of retreatment is the generation of cumulative toxicities. Bortezomib treatment produces different toxicities in Asians compared to Western populations, and bortezomib-based salvage therapy studies on Asians showed higher response rates compared to those performed on Western populations [11–13]. Bortezomib retreatment response rates and toxicities could also differ.

The purpose of this retrospective study was to evaluate the clinical efficacy and safety of bortezomib retreatment in patients with relapsed or refractory MM in Korea.

## 2. Patients and Methods

### 2.1. Patients

This study was a retrospective clinical trial conducted at the Chonnam National University Hwasun Hospital in Korea. Clinical records from November 2004 to April 2013 were reviewed. Eligibility criteria for the study included MM patients who underwent bortezomib retreatment after bortezomib-based salvage therapy, and patients had ≥1 measurable lesions at relapse. Measurable lesions were defined as (1) serum or urine M-protein, (2) measurable bone lesions or plasmacytomas, (3) measurable difference between involved and uninvolved FLC levels, and (4) bone marrow plasma cell percentage (absolute percentage must be ≥10%). We selected patients who relapsed or progressed ≥6 months after the last dose of the previous bortezomib therapy. Exclusion criteria included immunoglobulin M type MM, primary amyloidosis, and plasma cell leukemia.

### 2.2. Treatment

All patients received combination therapy during bortezomib retreatment. Treatment of Vel-CTD consisted of 3-week cycles of bortezomib (1.3 mg/m^2^ intravenously on days 1, 4, 8, and 11); dexamethasone (20 mg/m^2^/day intravenously on days 1, 4, 8, and 11); cyclophosphamide (150 mg/m^2^ orally on days 1 to 4); and thalidomide (50 mg orally daily for the entire 21 days) [13]. Vel-CD was the same regimen with the exclusion of thalidomide [12]. The start of a new cycle could be delayed on a weekly basis (for a maximum of 3 weeks) until recovery of toxicity (to a grade 2 or less) was achieved, allowing therapy to continue. If the patient had peripheral neuropathy of grade 2, or grade 1 with pain, bortezomib was reduced to 1.0 mg/m^2^; for ≥grade 3 peripheral neuropathy, bortezomib was withheld until the peripheral neuropathy resolved to baseline and then restarted at 0.7 mg/m^2^. Simultaneously, thalidomide was omitted until the toxicity resolved to baseline or decreased to below grade 1. Most of the patients were also given gabapentin (300–1,800 mg/day), nonsteroidal anti-inflammatory drugs, or opioids as adjuvants for pain control depending on pain severity. Since 2010, bortezomib was administered by weekly subcutaneous injection in patients with peripheral neuropathy of grade 2 or grade 1 with pain. Routine antiviral prophylaxis (acyclovir 400 mg once daily) for herpes zoster infection was administered [14] and patients in the Vel-CTD group were administered 100 mg aspirin to prevent deep vein thrombosis during thalidomide administration. All patients prophylactically received a proton pump inhibitor and monthly bisphosphonate treatment with zoledronate or pamidronate if they had lytic bone lesions. Thalidomide was discontinued permanently in the event of thrombosis despite prophylaxis [12].

### 2.3. Response and Toxicities Assessment

The myeloma response was evaluated during each cycle of bortezomib retreatment. After each cycle, the measurable disease was routinely checked including serum or urine M-protein, serum FLC and its ratio, and the size of plasmacytoma if physically possible. For evaluation of response the International Myeloma Working Group (IMWG) uniform response criteria were used [15], but there was no discrimination between complete response (CR) and stringent CR because the absence of clone cells in the bone marrow could not be confirmed in the retrospective data. Adverse events were graded for every cycle according to the National Cancer Institute's Common Terminology Criteria for Adverse Events (version 4.0, 2009).

### 2.4. Statistical Analysis

Descriptive statistics are summarized as frequency counts and percentages for categorical variables and as medians and ranges for continuous variables. The chi-square test was used to establish differences in the distribution of categorical data and Student's* t*-test to compare continuous variables. TTP was defined as the time from start of bortezomib retreatment to disease progression, with death from causes other than progression censored. Progression-free survival (PFS) was defined as the duration from start of bortezomib retreatment to disease progression or death, regardless of cause of death. Overall survival (OS) was defined as the duration from the start of bortezomib retreatment until the last follow-up or death [15]. TTP, PFS, and OS were analyzed using Kaplan-Meier survival curve estimates, and the differences between groups were compared using stratified log-rank tests. *P* < 0.05 was considered to indicate statistical significance, and 95% confidence intervals were calculated accordingly. All statistical computations were performed using the Statistical Package for the Social Sciences version 18.0 (SPSS, Chicago, IL, USA).

## 3. Results

### 3.1. Patients' Characteristics

Between November 2004 and April 2012, 165 patients received bortezomib-based salvage therapy and 34 of the 165 patients received bortezomib retreatment. Four patients were excluded because they received bortezomib retreatment within 6 months of the last dose of bortezomib salvage therapy. Therefore, 30 patients were enrolled in this study.

The baseline characteristics and disease demographics are described in Table 1. The median age at commencement of bortezomib retreatment was 67 years (range: 51–81) and 60% were ≥65 years. At the start of bortezomib retreatment 20% of patients were diagnosed with light-chain disease. The median time from diagnosis to bortezomib retreatment therapy was 43.6 months (range: 16.9–249.6) and the duration from initial last dose of bortezomib to retreatment was 12.9 months (6.7–63.6). At study enrollment 44% of patients were classified as Stage II or Stage III by the International Staging System (ISS). Patients had previously received the median number of 2 line chemotherapies (range: 2–5) and 50% of patients had previously received autologous stem cell transplantation (ASCT). Most of the patients had received prior thalidomide combination therapy (93.3%). For the bortezomib retreatment combination therapy regimen, 21 (70%) patients received We already defined the Vel-CTD and Vel-CD in methods, as reported previously [12]. Additionally 2 (6.7%) patients were enrolled in a clinical trial of dexamethasone and panobinostat combination therapy.

### 3.2. Treatment Response

The median number of treatment cycles was 6 (range: 2–12) and the number of cycles delivered was 196. For the best response after the initial bortezomib salvage chemotherapy, 73.3% of patients had CR, 10% had very good partial response (VGPR), 10% had partial response (PR), and 6.7% had SD. After the bortezomib retreatment therapies, 10 patients (33.3%) achieved CR, 2 patients (6.7%) had VGPR, and 6 patients (20%) had PR. The ORR (≥PR) was 60%. The relationship between best response to initial bortezomib therapy and bortezomib retreatment is shown in Table 1.

**Figure 1:**
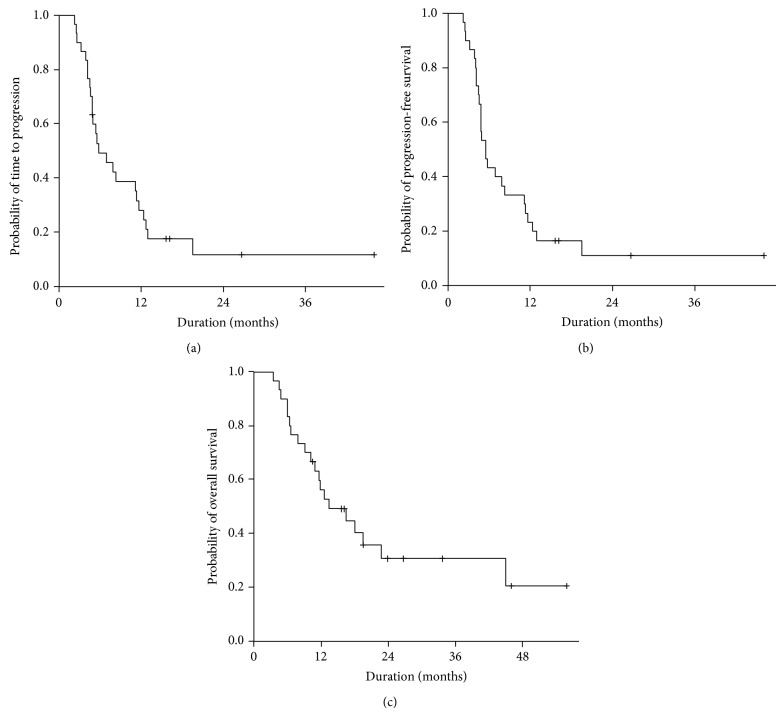
(a) Time to progression (TTP), (b) progression-free survival (PFS), and (c) overall survival (OS) of patients who received bortezomib retreatment. The median TTP was 5.8 months (95% CI: 2.6–9.0), median PFS was 5.8 months (95% CI: 4.2–6.8), and median OS was 13.4 months (95% CI: 6.1–20.7).

**Figure 2:**
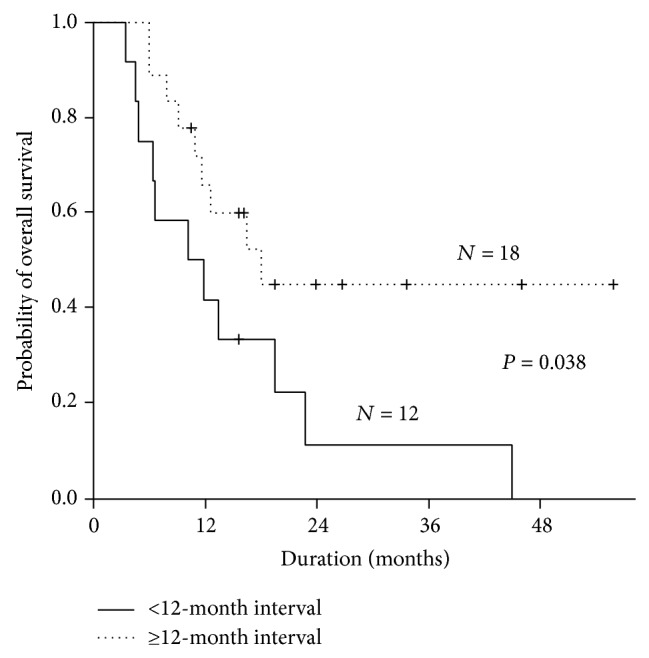
Overall survival (OS) according to time interval (months) between initial bortezomib-based therapy and retreatment. Patients who received bortezomib retreatment ≥12 months from the final initial therapy had a 1-year OS rate of 65.8% (95% CI: 43.5–88.1) and patients receiving retreatment after <12-month interval had a 1-year OS rate of 41.7% (95% CI: 13.9–69.5) (*P* = 0.038).

The median follow-up duration was 23.9 months (range: 10.5–55.8). The median TTP was 5.8 months [95% confidence interval (CI): 2.6–9.0], median PFS was 5.5 months (95% CI: 4.2–6.8), and median OS was 13.4 months (95% CI: 6.1–20.7; Figure 1). There was no significant difference in PFS or OS according to the combination therapy regimen with bortezomib retreatment (Vel-CD versus Vel-CTD) (*P* = 0.747). Survival was analyzed according to the time interval between the last dose of the initial therapy and retreatment with bortezomib. Eighteen patients commenced bortezomib retreatment with a ≥12-month interval and the remaining 12 patients commenced bortezomib retreatment with a <12-month interval. Of the patients who had a ≥12-month interval for bortezomib retreatment, the 1-year OS rate was 65.8% (95% CI: 43.5–88.1) compared to 41.7% (95% CI: 13.9–69.5) for patients with a <12-month interval (*P* = 0.038; Figure 2).

### 3.3. Adverse Events

During bortezomib retreatment, the most common adverse events were hematologic toxicities. Of the 30 patients, grade 3 or 4 hematologic toxicities including neutropenia (47%), thrombocytopenia (43%), leukopenia (37%), and anemia (10%) were observed (Table 3). Nonhematological toxicities were also observed (Table 2). The most frequently observed nonhematologic toxicity was asthenia (70%) and the second most common was sensory neuropathy (63%). The peripheral sensory neuropathy observed was grades 1, 2, and 3 in 33%, 27%, and 3% of patients, respectively. Gastrointestinal toxicity was mainly due to constipation; however, only 10% of patients experienced the maximal grade 2 constipation. Serious infection was observed in eight (27%) patients during bortezomib retreatment: pneumonia in five patients, fever in two, and bacteremia in one. No thromboembolic or bleeding episodes were observed during bortezomib retreatment.

A total of 17 (57%) patients experienced dose reduction from the originally planned bortezomib dosage. The median number of cycles for the start of bortezomib dose modification was 3 (range: 1–9). The change to weekly subcutaneous administration of bortezomib was observed in 13 patients and dosage reduction (from 1.3 mg/m^2^ to 1.0 mg/m^2^) in 4 patients. The causes of dose reduction were grade 2 sensory neuropathy in six patients and grade 2 asthenia in six patients. Another five patients received bortezomib dose modification because of diarrhea (grade 2), pain (grade 2), nausea (grade 2), neutropenia (grade 4), and poor compliance. Two patients received the weekly bortezomib administration schedule at the start of the first cycle because of grade 2 sensory neuropathy and concomitantly received gabapentin and opioids for pain control. Only two (7%) patients had interrupted bortezomib retreatment due to grade 3 sensory neuropathy and grade 3 asthenia. The majority of patients (70%) had the bortezomib retreatment interrupted due to disease progression, while 23% of patients achieved CR and received maintenance therapy thereafter.

## 4. Discussion

The purpose of this retrospective study was to evaluate the clinical efficacy and safety of bortezomib retreatment in patients with relapsed or refractory MM. The study included only patients who received bortezomib retreatment ≥6 months after the last dose of the initial treatment with bortezomib. All patients received three- or four-drug combination therapy and had also previously received bortezomib combined chemotherapy as a salvage treatment. The ORR was 60% and the median TTP, PFS, and OS were 5.8 months (CI: 2.6–9.0), 5.5 months (CI: 4.2–6.8), and 13.4 months (95% CI: 6.1–20.7), respectively. After initial bortezomib treatment, retreatment by later exposure to bortezomib was more effective in the control of progressed disease. Serious infection was observed in 27% of patients during bortezomib retreatment, and dose reduction was performed in 57% of patients. However, no patients died due to adverse events during bortezomib retreatment.

Petrucci et al. [6] reported a phase II study of bortezomib retreatment in patients with relapsed MM. This prospective study included 130 patients and demonstrated an ORR of 40%. There were two main differences between this prospective study and the retrospective study reported here: patients in the prospective trial received bortezomib only or bortezomib-dexamethasone combination therapy and 30% of patients in the prospective study received stem cell transplantation, compared to 50% in our cohort. Based on these results, multiple drug combination in bortezomib retreatment is considered an effective and relatively safe method, even if patients were previously exposed to bortezomib. In the phase III VISTA trial, 77 (22%) patients received subsequent bortezomib and/or bortezomib combined retreatment after the first-line therapy (bortezomib, melphalan, and prednisone; VMP), and an investigator-assessed response rate of 50% was observed [16]. The VISTA trial used the VMP regimen for the initial first-line therapy and this might be the reason for the higher ORR compared to the phase II prospective trial [6, 16].

Two retrospective studies have included relatively large numbers of patients and showed marked differences in the best response to retreatment with bortezomib [8, 9]. Conner et al. [8] reported that only 21% of patients showed at least a PR to bortezomib retreatment. However, 41% of their cohort were nonresponders who did not achieve at least a PR to initial bortezomib treatment, and this study defined the gap between the final dose of the initial treatment and retreatment as only 60 days. Among patients for whom response assessment was available for both treatments, 32% of initial bortezomib responders had at least a PR to retreatment. Hrusovsky et al. [9] enrolled only patients who achieved at least a PR in previous bortezomib treatment and included all patients regardless of the duration from the end of initial treatment to the start of bortezomib retreatment. The ORR to bortezomib retreatment was 63.3%, and the patients with a ≥6-month interval from the last dose of initial bortezomib had an ORR of 76.9%. The major reason for the difference in ORR after bortezomib retreatment between the two retrospective studies is that Hrusovsky et al. [9] included only patients who showed an initial response to bortezomib therapy. Our patients were observed the higher CR rate (33.3%) than the other Caucasian data (0–13.3%) like that previous study of salvage bortezomib treatment results in Korean. However, there were no differences in the TTP or OS between our Korean data and Caucasian data [6, 8, 9, 12]. While our study enrolled only patients who received bortezomib retreatment after ≥6 months from the last dose of initial bortezomib treatment, the ORR was lower than that reported by Hrusovsky et al. [9]. Our cohort could be considered a heavily treated group because 50% of patients received the ASCT despite being of similar age, most patients were exposed to thalidomide (93%), and all patients received cyclophosphamide and dexamethasone combination therapy during initial bortezomib treatment.

Dose-limiting peripheral neuropathy is the major toxicity caused by bortezomib. Retreatment with bortezomib showed similar rates of sensory neuropathy when compared to initial bortezomib therapy [12]. While 70% of patients received the Vel-CTD regimen, grade 3 or more sensory neuropathy was rare in this study compared to our previous report [12]. Approximately 40% of patients received bortezomib dose adjustment due to treatment toxicity in the initial bortezomib therapy study, and 17 (57%) patients underwent dose reduction from the planned bortezomib dosage in this study [12]. Early modification of bortezomib administration to the weekly subcutaneous regimen may have contributed to the reduction of ≥grade 3 sensory neuropathy, even though the combination of thalidomide and bortezomib was used. Grade 4 hematologic toxicities were frequently observed, especially in neutropenia (17%) and thrombocytopenia (20%). However, there was no difference in the incidence of pneumonia or grade 4 hematologic toxicities in the initial bortezomib therapy, and only two (7%) patients had disrupted bortezomib retreatment due to sensory neuropathy and asthenia in this study [12]. The major cause of bortezomib retreatment cessation was disease progression (70%). The major concern for retreatment with bortezomib is drug resistance and toxicity, and this could be relatively controllable with vigorous monitoring of toxicities and modification of dosage. The recent meta-analysis reported the grade 3/4 toxicities in patients with bortezomib retreatment as 4.2% of peripheral neuropathy, 16.9% of neutropenia, and 37.6% of thrombocytopenia [17]. This meta-analysis mostly included the Caucasian and the hematologic toxicities were less frequently observed in comparison with our Korean data. However, there was no difference in grade 3/4 sensory neuropathy (4.2% versus 3%) between our Korean data and Caucasian data. The analysis of the OS according to the interval between initial therapy and retreatment revealed that bortezomib retreatment after ≥12 months from the last dose of initial therapy provided a significant survival benefit. This suggests that the sensitive relapse group could be selected using the time interval between initial bortezomib therapy and retreatment. The limitation of this study for generalization of the efficacy and safety of bortezomib retreatment is that this was a retrospective analysis of a small number of patients enrolled in a single institute. However, this study has clinical significance due to the scarcity of clinical data regarding the effectiveness of bortezomib retreatment with regard to response and toxicities, especially in the Asian population.

In conclusion, this study suggests that retreatment with bortezomib is an effective strategy for patients who relapsed with a long interval after initial bortezomib therapy. The data support the safety of bortezomib retreatment with active dosage modification.

## Figures and Tables

**Table 1:** Patients' characteristics for bortezomib retreatment.

Characteristic	Patients, *n* (%)
Total number of patients	30
Gender, male	10 (33.3)
Median age at start of bortezomib retreatment, years (range)	67 (51–81)
Age ≥65 years, *n* (%)	18 (60)
Median time in months from diagnosis to bortezomib retreatment (range)	43.6 (16.9–249.6)
Median time in months from initial bortezomib treatment to retreatment (range)	12.9 (6.7–63.5)
Paraprotein type at bortezomib retreatment	
IgG	17 (56.7)
IgA	7 (23.3)
Light-chain disease	6 (20.0)
Durie-Salmon stage at diagnosis, *n* (%)	*n* = 28
I	4 (14.3)
II	6 (21.4)
III	18 (64.3)
Creatinine ≥2 mg/dL at diagnosis	4 (13.3)
ISS stage at diagnosis (%)	*n* = 28
I	12 (42.9)
II	6 (21.4)
III	10 (35.7)
ISS stage at the study enrollment (%)	*n* = 25
I	14 (56.0)
II	5 (20.0)
III	6 (24.0)
Prior treatments, median (range)	2 (2–5)
Prior line of therapy, *n*	
2	17 (57)
3	6 (20)
4	4 (13)
5	3 (10)
Prior autologous stem cell transplantation, *n* (%)	15 (50.0)
Prior thalidomide exposure	28 (93.3)
Prior bortezomib combination therapy	
Vel-CD	10 (33)
Vel-CTD	20 (67)
Combination chemotherapy during bortezomib retreatment	
Vel-CD	7 (33.3)
Vel-CTD	21 (70)
Velcade, dexamethasone, panobinostat	2 (6.7)

ISS: International Staging System; Vel-CD: bortezomib, cyclophosphamide, and dexamethasone; Vel-CTD: bortezomib, cyclophosphamide, thalidomide, and dexamethasone; FISH: fluorescence in situ hybridization; CR: complete response; VGPR: very good partial response; PR: partial response; SD: stable disease; PD: progressive disease.

**Table 2:** Relationship between best response to initial bortezomib treatment and best response to bortezomib retreatment.

		Best response to bortezomib retreatment					
		ORR (%)	CR	VGPR	PR	SD	PD
Best response to initial bortezomib treatment	ORR (%)	30	10 (33)	2 (7)	6 (20)	10 (33)	2 (7)
	CR	22 (73)	9	2	5	4	2
	VGPR	3 (10)	0	0	1	2	0
	PR	3 (10)	1	0	0	2	0
	SD	2 (7)	0	0	0	2	0

ORR: overall response rate; CR: complete response; VGPRL very good partial response; PR: partial response; SD: stable disease; PD: progressive disease.

**Table 3:** Hematologic and nonhematologic toxicities during bortezomib retreatment therapy according to the NCI-CTCAE 4.0.

Grade	Number of patients in total 30, *n* (%)			
	1	2	3	4
Leukopenia	5 (17)	9 (30)	8 (27)	3 (10)
Neutropenia	3 (10)	8 (27)	9 (30)	5 (17)
Anemia	8 (27)	15 (50)	3 (10)	0
Thrombocytopenia	5 (17)	10 (33)	7 (23)	6 (20)
Creatinine	2 (7)	3 (10)	1 (3)	0
Anorexia	8 (27)	0	0	0
Nausea	6 (20)	1 (3)	0	0
Vomiting	1 (3)	1 (3)	0	0
Diarrhea	2 (7)	3 (10)	0	0
Constipation	5 (17)	3 (10)	0	0
Dyspnea	3 (10)	1 (3)	0	0
Pneumonia	0	0	5 (17)	0
Fever/bacteremia	0	0	3 (10)	0
Sensory neuropathy	10 (33)	8 (27)	1 (3)	0
Motor neuropathy	4 (13)	1 (3)	1 (3)	0
Pain	10 (33)	4 (13)	0	0
Dizziness	11 (37)	0	0	0
Delirium	1 (3)	0	0	0
Insomnia/somnolence	6 (20)	0	0	0
Asthenia	9 (30)	11 (37)	1 (3)	0
Rash	2 (7)	0	0	0

NCI-CTCAE: National Cancer Institute Common Terminology Criteria for Adverse Events.
